# The Nucleolus: In Genome Maintenance and Repair

**DOI:** 10.3390/ijms18071411

**Published:** 2017-07-01

**Authors:** Maria Tsekrekou, Kalliopi Stratigi, Georgia Chatzinikolaou

**Affiliations:** 1 Institute of Molecular Biology and Biotechnology, Foundation for Research and Technology-Hellas, Nikolaou Plastira 100, 70013 Heraklion, Crete, Greece; mtsekre@imbb.forth.gr (M.T.); callina@imbb.forth.gr (K.S.); 2 Department of Biology, University of Crete, Vassilika Vouton, 71409 Heraklion, Crete, Greece

**Keywords:** nucleolus, DNA repair, nucleus, ribosomal DNA

## Abstract

The nucleolus is the subnuclear membrane-less organelle where rRNA is transcribed and processed and ribosomal assembly occurs. During the last 20 years, however, the nucleolus has emerged as a multifunctional organelle, regulating processes that go well beyond its traditional role. Moreover, the unique organization of rDNA in tandem arrays and its unusually high transcription rates make it prone to unscheduled DNA recombination events and frequent RNA:DNA hybrids leading to DNA double strand breaks (DSBs). If not properly repaired, rDNA damage may contribute to premature disease onset and aging. Deregulation of ribosomal synthesis at any level from transcription and processing to ribosomal subunit assembly elicits a stress response and is also associated with disease onset. Here, we discuss how genome integrity is maintained within nucleoli and how such structures are functionally linked to nuclear DNA damage response and repair giving an emphasis on the newly emerging roles of the nucleolus in mammalian physiology and disease.

## 1. Introduction

To sustain life, cells must be able to maintain and appropriately utilize their genome with the ultimate goal to produce the multitude of macromolecules ranging from small non-coding RNAs to large multisubunit proteins. Unlike RNAs, however, proteins require that cells allocate most of their energy reservoirs to generate functional ribosomes, the ribonucleoprotein factories where proteins are synthesized. It is, therefore, not surprising that ribosome synthesis represents a universal and continuous process for all living systems. A fundamental role in this process is held by the nucleolus, where ribosomal RNA is synthesized and ribosomal subunits are assembled. Though nucleoli are not visible during distinct phases of the cell cycle or specific differentiation stages, it is commonly accepted that any cell devoid of its nucleoli is either deceased or dying [1].

Under the microscope, the nucleolus is often the most prominent sub-nuclear formation of almost every eukaryotic cell during interphase. Consequently, it was described as early as 1781 by Fontana [2] and extensively studied for over a century [3]. By the end of the 19th century, researchers had ended up with conclusions that are still valid [4]. The number of visible nucleoli per cell varies enormously depending on cell cycle, cellular activity or differentiation status. For example, nucleoli are not visible in spermatozoa or in eukaryotic cells that undergo open mitosis (in open mitosis the nuclear envelope is completely disassembled). Moreover, nucleoli tend to be bigger in large cells and present in bigger numbers in growing cells [5]. The nucleolus forms at specific chromosomal loci [6,7,8] that are called “secondary constrictions” (the primary constriction is the centromere) or “nucleolar organizer regions” (NORs). NORs were found to consist of tandem arrays of ribosomal genes (rDNA) that encode 18S, 28S, and 5.8S ribosomal RNAs (rRNAs) [9,10,11,12] and the nucleolus was established as the site of ribosome biogenesis [13,14].

For three decades, research was focused on delineating the mechanisms that govern ribosomal synthesis and assembly. Recent evidence, however, suggests that the nucleolus is a multifunctional organelle. For instance, a large—if not the greatest—number of proteins in the nucleolus have functions that are not directly linked to ribosomal biogenesis [15,16,17]. Moreover, the presence of numerous RNA modifying enzymes in the nucleolus suggests that many non-ribosomal RNA post-transcriptional modifications take place in this organelle. In fact, all snRNAs localize transiently to the nucleolus to be modified by 2’-*O*-methylation and pseudouridylation [18]; interestingly, RNase P subunits and its RNA component are localized in the nucleolus suggesting that 5’ processing of tRNA might take place here [19,20,21]. Furthermore, the RNA component of Signal Recognition Particle and some of its subunits have a nucleolar biogenesis phase [22,23,24]. Other proteins, such as cell cycle regulatory factors, DNA repair enzymes or signaling kinases, are also known to reside in the nucleolus. Being the center of ribosomal biogenesis, the nucleolus provides a suitable territory to integrate environmental and intracellular signals, such as various stress stimuli ranging from nutrient deprivation and rapid pH fluctuations to genotoxic stress, in order to fine-tune complex biological processes.

In humans, rDNA represents only 0.4% of the genome and is found in low copy numbers within nucleoli, thus, any rDNA insults are expected to represent a minor fraction of total nuclear DNA damage. However, rDNA is considered one of the most unstable genomic sites [25]; the repetitive nature of rDNA renders it prone to improper recombination that may often lead to deletions or rearrangements. Moreover, the high transcription rate of rDNA is thought to cause a great amount of topological stress as well as R-loops, the three-stranded nucleic acid structures that are composed of a RNA:DNA hybrid and the associated non-template single-stranded DNA [26]; if not properly restored, R-loops are thought to generate DSBs [27]. Here, we discuss the processes that surveil genome integrity within nucleoli, how DNA damage affects nucleolar morphology and function and how a stressed nucleolus might affect disease onset and cell viability.

## 2. Nucleolus and the rDNA: Structure, Function and Organization

The nucleolus is a self-organizing membrane-less “organelle” which is “formed by the act of building a ribosome” [28]. Indeed, the level of ribosome production is reflected on the size of the nucleolus [29] and the molecular processes arising in this organelle determine its structural organization [30]. In higher eukaryotes, electron microscopy reveals that during interphase nucleoli sustain a tripartite structure: the fibrillar center (FC), the dense fibrillar compartment (DFC), and the granular center (GC). FCs contain inactive rDNA with the potential to be directly activated and proteins of the transcription machinery, such as RNA polymerase I, Topoisomerase I and Upstream Binding Factor (UBF) [31,32,33]. The DFC surrounds the FCs and consists of pre-rRNA and early processing factors [34], while at the GC, late processing factors and ribosomal proteins reside and ribosomal assembly takes place [35]. It is now broadly accepted that transcription takes place at the boundary of the FC and the DFC; instead, rRNA processing begins at the DFC and is completed in the GC.

Interestingly, in many eukaryotic cells, the nucleolus becomes disorganized when the cell enters mitosis and transcription is inhibited [36]. Despite nucleolar disorganization, UBF and DNA topoisomerase I remain associated with the NORs. In contrast, proteins from the DFC and the GC, such as nucleophosmin and fibrillarin, dissociate from the nucleolus, although these factors still remain associated with the perichromosomal layer during metaphase and anaphase; in doing so, these proteins form prenucleolar bodies during telophase, before being recruited to the newly formed nucleoli. Depending on the cell type, the nucleolus reappears when RNA polymerase I associates again with one NOR during late anaphase or telophase. In G1, multiple nucleoli may then fuse to form larger nucleoli containing several NORs (reviewed in [37]). The yeast uses a closed mitosis model (the nuclear envelope remains intact); as such, nucleoli are not disrupted during mitosis and are visible throughout the cell cycle [38].

**Figure 1:**
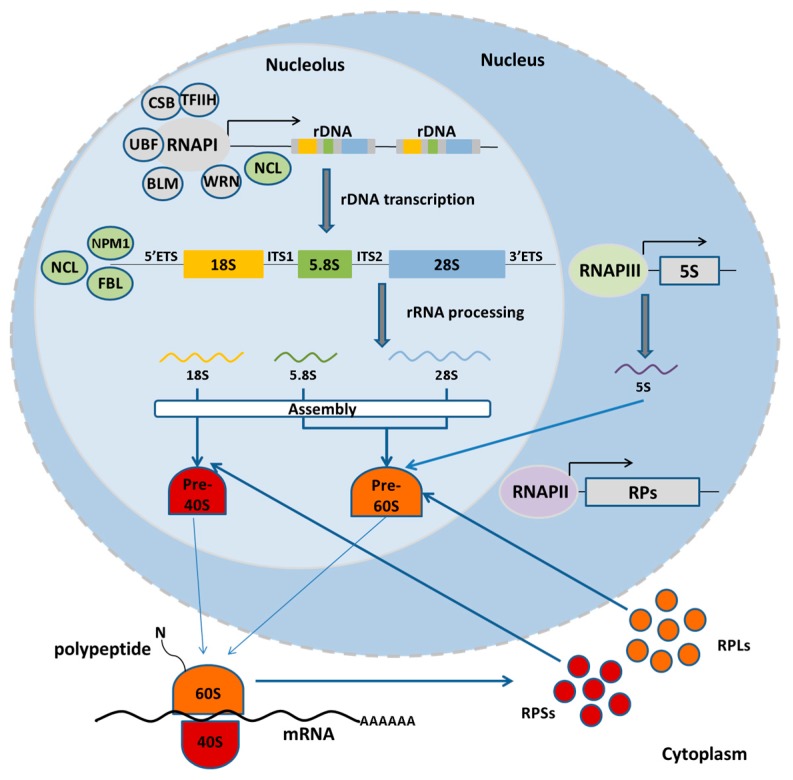
The function of nucleolus. In normal state, the RNA Pol I machinery transcribes a polycistronic pre-rRNA molecule, which is subsequently processed to mature rRNAs (18S, 5.8S, and 28S) of the small and large ribosomal subunit. Outside the nucleolus, RNA Pol III transcribes the fourth rRNA (5S), while RNA Pol II transcribes the ribosomal protein genes to be translated in the cytoplasm. Ribosomal proteins of the large (RPLs) and the small (RPSs) subunit enter the nucleolus to associate with rRNAs and assemble the ribosomal subunits, which are then exported to the cytoplasm.

There are four rRNA genes, namely the small subunit S-rRNA (18S), the 5.8S rRNA, the large subunit L-rRNA (25S/28S) and the 5S rRNA. The first three are transcribed as a single precursor rRNA molecule—in the order of S-rRNA, 5.8S rRNA and L-rRNA—by RNA polymerase I, which is then processed to remove the 5’ external transcribed spacer (5’ETS), the two internal transcribed spacers (IGS) flanking the 5.8S rRNA and the 3’ external transcribed spacer (3’ETS). The rDNA repeats are separated by non-transcribed intergenic spacer regions (ITS) [39,40] (Figure 1). Whereas the rRNA genes are highly conserved among species, the various intergenic and transcribed spacer regions are highly divergent [1]. The fourth rRNA (5S rRNA) is also organized in tandem arrays but it is transcribed by RNA polymerase III outside the nucleolus. In yeast, the 5S rDNA is found in the same transcription unit as the other rRNA genes but it is transcribed in the opposite direction by RNA polymerase III. The number of rDNA repeats varies enormously throughout phylogeny. Birds and mammals have 100–300 copies per haploid genome, while amphibians and plants may have thousands of copies [40]. Humans and mice bear ~200 rRNA copies per haploid genome. In humans, rRNA genes are located between the short arm and the satellite body of acrocentric chromosomes 13, 14, 15, 21, and 26 [41]. In mice, rDNA clusters are found within the centromeric regions of chromosomes 12, 15, 16, 18, and 19 [42,43]. In *Saccharomyces cerevisiae*, the rRNA genes are located on the right arm of chromosome XII in a tandem array of 150–200 copies [44]. The rDNA can also be differentially amplified as in polytene chromosomes in Drosophila salivary gland nuclei or even extrachromosomally as in amphibian oocytes [1].

The structural and catalytic subunit of ribosomes is composed of rRNA, which, unlike its protein counterparts, cannot be amplified by translation. Instead of investing in higher transcription rates for rDNA or in maximizing ribosomal RNA stability, cells possess a large number of rRNA genes to sustain the high demand for ribosomes. Nevertheless, few hundred rRNA gene copies appear to be enough to meet this requirement, especially when one considers that only a fraction of ribosomal genes is routinely transcribed. Indeed, in yeast, flies or mammals, only 50% of the rRNA gene copies are transcribed; in line, mutant model organisms with less than half of the copies are viable [45,46,47]. Although, at present, such a highly conserved redundancy remains hard to explain, it is possible that distinct cell types or specific developmental stages require the rapid increase in protein synthesis. Moreover, Kobayashi has proposed that rDNA may act as a DNA damage sensor activating DNA repair or else triggering apoptosis [25] (further discussed in Section 6).

The formation and dissolution of nucleoli during cell cycle progression raised the question of what are the essential requirements for nucleolar formation. Subsequent studies revealed UBF, a transcription factor that recognizes the ribosomal RNA gene promoter, as a key factor in this process, as it binds excessively on human rDNA copies throughout the cell cycle. Indeed, a series of experiments with pseudoNORs, that contained megabase-long synthetic UBF-binding site repeats and were inserted into non-acrocentric human chromosomes, provided the first evidence that binding of UBF to specific DNA sites is required for the appearance of active NORs [48]. During metaphase, pseudoNORs form subnucleolar structures with a protein composition that is similar to that seen in endogenous active NORs. PseudoNORs, however, do not contain promoters or pre-rRNA-coding sequences and, thus, they do not form nucleoli during interphase. Further evidence for the pivotal role of UBF in nucleolar formation was given by neoNORs. Grob et al. constructed artificial NORs by combining UBF-binding site arrays with human promoters that drive the transcription of mouse pre-rRNAs. NeoNORs do form functional neonucleoli, organized in FCs, DFCs and GCs and produce functional ribosomal subunits that can be found in polysomes [49]. In essence, UBF binding to DNA, along with active rDNA transcription, is sufficient to form a nucleolus in human cells. In line, UBF marks the presence of active NORs throughout the cell cycle; in doing so, it remains attached to those NORs that were active during the previous interphase to ensure the rapid re-initiation of transcription. This strategy appears to be highly conserved, as UBF is present across all animal phyla.

## 3. The Nucleolus Associated Heterochromatin and Its Role in Safeguarding Genome Integrity

During interphase, each nucleolus is typically surrounded by heterochromatin. A subset of rDNA loci is transcribed by looping inside the nucleolus. Instead, inactive rDNA genes are associated with the nucleolar periphery and are characterized by repressive marks of constitutive heterochromatin [50,51]. Using next generation sequencing technologies, two independent studies have recently identified the nucleolus-associated chromatin domains (NADs), that is the DNA derived from isolated nucleoli [52,53]. Both studies identified similar NAD genomic regions containing apart from rDNA, also satellite, centromeric and pericentromeric DNA, silent chromatin and the absence of genes.

It is thought that the heterochromatin at rDNA repeats may play a role in regulating rRNA transcription. In mammalian cells, rDNA exists in an active, silent or stable silent chromatin state. Active rDNA genes are typically not CpG-methylated or associated with nucleosomes [54] and reside at the boundary of the FC and the DFC. The silent chromatin is organized in nucleosomes bearing histone repressive marks; it contains non-methylated DNA and is found at the center of FCs. Instead, stable silent rRNA genes acquire all heterochromatin marks, both histone repressive marks and DNA CpG methylation. This range of modifications likely allows for the fine-tuning of transcription regulation and for the control of rDNA stability. When rRNA synthesis needs to be minimized, rRNA genes acquire silent histone modifications but not CpG methylation; this is a silent chromatin state that can easily be reversed when necessary. In contrast, stable silent rDNA cannot easily be reverted owing to extensive CpG methylation [55,56,57].

The evolutionarily conserved organization of a fraction of rDNA into heterochromatin seems to be important not only for nucleolar structure and regulation of rDNA transcription, but also for safeguarding genomic stability by rendering heterochromatic rDNA less accessible to intrinsic genotoxic byproducts of metabolism, or to the cellular DNA recombination machinery. In mammals, switching rDNA between active and inactive state is mediated by the nucleolar remodeling complex (NoRC) that belongs to the ATP-dependent SNF2h family. Transient deletion of its large subunit TTF-1-interacting protein-5 (TIP5) [56,58,59] results in loss of heterochromatin, rDNA instability, nucleolar disorganization and cellular senescence [25,60], implying that rDNA silencing plays an important role in maintaining rDNA integrity. Loss of CpG methylation, by DNMT1 or DNMT3b inactivation or using the DNA methylation inhibitor aza-dCIn, reactivates silent rRNA genes and results in rDNA instability as evident by the formation of episomal rDNA circles [61]. In Drosophila, deletion of the histone methyltransferase Su(var)3-9, responsible for establishing H3K9me2 in constitutive heterochromatin, results in global heterochromatin disruption, rDNA instability and formation of extrachromosomal rDNA circles [60]. It cannot be distinguished, however, whether rDNA instability is caused directly by heterochromatin disruption or is the consequence of global genome instability.

## 4. Genome Maintenance inside the Nucleolus

rDNA is considered a highly fragile genomic entity. High transcription rates cause topological stress due to the extensive formation of RNA:DNA hybrids. If not readily dissolved in a timely manner, such structures may trigger cytotoxic DNA double strand breaks (DSBs). Topoisomerases, the enzymes that participate in the over- or un-winding of DNA, are known to relax this topological stress. In support, inhibition of topoisomerases is known to induce DSBs in rDNA or other highly transcribed sites [62,63,64]. Moreover, the repetitive nature of rDNA renders it particularly prone to homologous recombination events; i.e., DNA repeats within the same or between different clusters inappropriately recombined, which triggers either chromosomal translocations or the loss/amplification of rDNA. Interestingly, the striking copy number variation between individuals or even within cells of the same individual, suggests a high level of meiotic (>10%) and mitotic recombination frequency in the rDNA locus [65]. Moreover, increased mitotic instability of the rDNA locus is a trait of more than half of lung and colorectal adult cancer samples as detected by rDNA rearrangements, relative to the surrounding normal tissue or the peripheral blood of each patient [66]. Indeed, the recently identified presence of palindromic rDNA repeats in prematurely aging WRN patients [67], the abnormal nucleolar morphology of cancer cells as well as the cancer prone phenotype of certain ribosomopathies, suggest that rDNA instability may be, in several occasions, causal to premature disease onset and/or progression, including cancer. In this section, we discuss the mechanisms that maintain genome stability within the nucleolus, with a focus on DSB repair and Nucleotide Excision Repair. Repair of smaller non-helix distorting lesions by Base Excision Repair and Mismatch Repair is not included in this review, as the contribution of these pathways in nucleolar genome maintenance has not yet been evidenced.

### 4.1. DSB Repair and the Nucleolus

DNA DSBs are highly cytotoxic lesions that interfere with transcription and DNA replication, triggering large chromosomal rearrangements and threatening cell survival. Similar to every other genomic site, persistent R-loops, endogenously generated free radicals and exposure of rDNA to ionizing radiation (IR) or genotoxins e.g., bleomycin or zeocin may trigger DSBs. Eukaryotic cells have evolved two main pathways to resolve DSBs, the non-homologous end joining pathway (NHEJ) and the homologous recombination pathway (HR). In mammalian cells, NHEJ is active throughout the cell cycle; instead, HR is active in late S and G2 phases when the sister chromatid provides the homologous template required for repair DNA synthesis.

**Figure 2:**
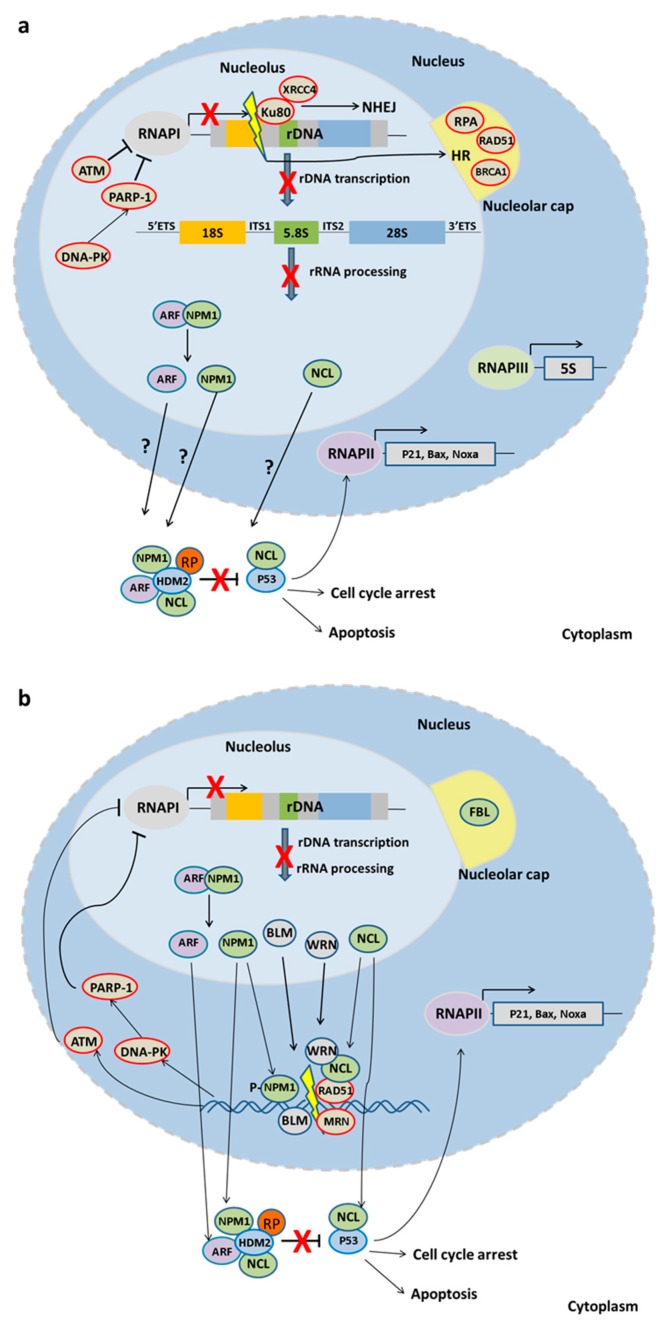
The nucleolar response to DNA damage. (**a**) DSBs in the rDNA cause nucleolar segregation and nucleolar cap formation. While DSBs outside the nucleolus result in a global nuclear response, DNA damage inside the nucleolus elicits only a localized response. DNA damage kinases, ATM and DNA-PK, inhibit RNA Pol I to cease transcription and subsequent rRNA processing and ribosomal assembly. Ribosomal proteins and possibly nucleolar proteins activate the P53 pathway. Damaged DNA is predominantly repaired by NHEJ, while HR, when activated, contributes to rDNA instability. (**b**) DSBs outside the nucleolus also induce nucleolar segregation and cap formation. RNA Pol I is inhibited by ATM and DNA-PK kinases while nucleolar proteins translocate to the damaged site in the nucleoplasm to participate in DNA repair as well as to the cytoplasm to activate the stress response.

In mammalian cells, rDNA breaks cause nucleolar caps; these are unique structures at the nucleolar periphery that contain individual, most probably damaged, NORs [68]. Similar to the relocalization of rDNA DSBs to nucleolar caps, DSBs outside the nucleolus often cluster together [69,70], likely to facilitate HR repair through homologous pairing [71,72]. Accordingly, DSBs in heterochromatic DNA relocate outside heterochromatin for accessibility of repair factors [73,74]. Interestingly, approximately half of the rDNA repeats are silent and localized at the perinucleolar heterochromatin. rDNA DSBs relocate to nucleolar caps, probably independently of the chromatin state where the lesion occured [68], which could be considered a consensus mechanism of DSB relocation for repair in any genomic locus. In yeast, DNA DSBs also relocate to the periphery of the nucleolus, but unlike mammalian cells, the nucleolus does not segregate [75]. Nucleolar segregation is a consequence of DNA-dependent protein kinase (DNA-PK) and/or Ataxia-telangiectasia mutated (ATM) dependent transcription inhibition. In mouse cells, ATM inhibits RNA polymerase I by interfering with the formation of the pre-initiation complex, leading to the premature displacement of elongating RNA polymerases [76,77]. On the contrary, studies in human cells show that rDNA silencing upon exposure to IR occurs in a DNA-PK-dependent manner [78]; the latter recruits PARP1 to damaged chromatin sites to cease ongoing transcription. DNA-PK has also been shown to inhibit RNA polymerase I in vitro by blocking the formation of the pre-initiation complex [79,80] (Figure 2a). Even so, both kinases are able to contribute in post-DNA damage associated rDNA silencing through the (in)direct phosphorylation of nucleolar proteins involved in transcription and rRNA processing [81,82,83,84].

To determine whether rDNA DSBs are repaired by NHEJ or HR, three recent studies have employed the *Physarum polycephalum* endonuclease I-Ppol that recognizes a 15-base pair site within the 28S coding sequence to induce DSBs within rDNA. Harding et al. identified NHEJ as the predominant DSB repair pathway in the nucleolus [85]. Unlike the depletion of HR factors, inhibition of DNA-PK or the transient depletion of NHEJ factors triggered the accumulation of DSBs upon I-Ppol induction. The exact site of NHEJ repair has not yet been clarified; Ku80, DNA-PK and XRCC4 are detected in the nucleoli but are absent in nucleolar caps, suggesting that these factors associate with DNA lesions within nucleoli [86]. Instead, van Sluis et al. showed that HR is also involved in the DSBs repair of rDNA [68]. HR takes place in nucleolar caps that stain positive for rDNA, γH2AX and distinct HR proteins, such as BRCA1, RPA2 and Rad51. In addition, unscheduled DNA synthesis was detected in nucleolar caps, indicating repair by DNA synthesis. HR as well as unscheduled DNA synthesis in nucleolar caps, occurred also in G1, suggesting that HR in rDNA happens in the absence of a sister chromatid, possibly by using other rDNA repeats as a template. A drawback of the methodology used, however, may be that the I-Ppol recognition site is found in every rDNA repeat. Thus, both studies were likely performed under an unusually high number of rDNA DSBs. Warderdam et al. investigated the time course of rDNA breaks resolution and concluded that DNA damage is repaired by NHEJ independently of the break source, I-Ppol or IR; in fact, HR appears to impede proper repair and when the latter DNA repair mechanism is active, it results in the reduction of rDNA repeats [87]. Indeed, rDNA HR mediates an error-prone repair process that causes rDNA instability. In essence, the presence of DSBs in nucleoli triggers an ATM- and/or DNA-PK-dependent rDNA silencing leading to nucleolar segregation and cap formation. NHEJ is likely the predominant repair pathway, while HR takes place in a cell cycle independent manner and possibly results in rDNA repeat reduction. Interestingly, the response to rDNA DSB in nucleoli is also unique; unlike the pan-nuclear response of cells to DSBs, in nucleoli ATM is activated locally and transcription inhibition is restricted only to specific rDNA sites [68,76].

### 4.2. Nucleotide Excision Repair and the Nucleolus

Nucleotide excision repair (NER) is a versatile DNA repair pathway that resolves a variety of helix distorting lesions, such as bulky adducts and UV-induced cyclobutane pyrimidine dimers (CPDs) and 6-4 photoproducts, which impede transcription and DNA replication. NER is divided into two sub-pathways: the global genome repair (GG-NER), which resolves DNA lesions throughout genome, and the transcription-coupled repair (TC-NER) that is directed on the transcribed strand of active genes. The two NER subpathways differ in how the lesion is recognized, with XPC-RAD23 being specific for GG-NER whereas, during TC-NER, CSA and CSB factors are recruited at the DNA damage-stalled RNA Pol II; both subpathways then merge to a common repair machinery [88].

Although TC-NER factors are localized in nucleoli, a number of studies have shown that TC-NER is not active in rDNA loci whereas GG-NER may be hampered by the structural compartmentalization of rDNA, evident by the absence of XPC in the nucleolus [89]. In hamster cells, CPDs remained unresolved in the rDNA locus even 24 h post UV irradiation [90,91]. Both NER subpathways are also inactive in mouse rDNA [92]. In human cells, CPDs are removed from rDNA at a very slow rate, both in growing and quiescent cells, implying that CPD repair occurs independently of transcription. On the other hand, in the absence of functional TC-NER (CSA or CSB), CPD removal was substantially reduced compared to repair-proficient cells, whereas cells deficient in GG-NER showed no repair of CPDs [93], implying a role for both GG- and TC-NER in repairing the rDNA locus. Conclusively, how and to what extent NER factors contribute to the resolution of UV-induced damage remains unclear, though it has been proposed that rDNA is likely maintained by recombination instead of TC-NER [91]. This may be further supported by recent findings that implicate CSB in Transcription-Coupled Homologous Recombination of oxidatively induced DSBs [94], though such a mechanism has not yet been described, for neither the nucleolus nor UV-induced lesions. Recently, CSB has also been shown to participate in repair of nucleolar oxidative lesions [89]. Interestingly, unlike DSB formation that can be targeted to the nucleolus by employing sequence-specific nucleases such as I-Ppol, exposure to UV irradiation introduces lesions to the whole nucleus and elicits the DNA damage response that may potentially impede rDNA repair due to nucleolar re-organization upon stress.

## 5. The Nucleolus upon Stress

Being the hub integrating environmental and intracellular signals, the nucleolus undergoes dramatic structural changes upon DNA damage. When cells are exposed to damage, for example UV or IR, the FC and the GC condense and separate from the nucleolar body resulting in the so-called nucleolar segregation. This is accompanied by proteins moving from nucleoli to the nucleoplasm and vice versa.

It is now established that any kind of stress that impairs any step of ribosomal biogenesis, i.e., rDNA transcription, rRNA processing, ribosomal protein synthesis and the ribosomal subunits assembly, causes nucleolar disruption and subsequently activates P53-dependent response [95,96,97,98], resulting in cell cycle arrest or apoptosis. P53-independent nucleolar stress response has also been described in yeast, that lacks P53, and in Drosophila, that lacks a Double minute 2 (MDM2) homologue [98]. In mammals though, such a pathway remains either silent or not yet uncovered. In the nucleus, P53 upregulates transcription of cell cycle (e.g., *p21*) and apoptotic regulators (e.g., *Bax*, *Noxa*) transcribed by RNA pol II, whereas in the cytoplasm P53 activates the mitochondrial apoptotic pathway [99,100,101]. P53 may also inhibit RNA Pol I transcription by disrupting the interaction between the basal transcription factors SL1-UBF, which results in a decrease in ribosome subunit biogenesis [102]. Under normal physiological conditions, the P53 levels are kept low: the interaction with E3 ubiquitin ligases, such as HDM2 for human and MDM2 for mouse, ensures the P53 export from the nucleus (monoubiquitinylation) and degradation (polyubiquitinylation) or inhibition of its transactivation domain. Upon cellular stress (e.g., DNA damage, pH fluctuation, heat shock or nutrient deprivation), the HDM2-P53 interaction is disrupted, thus activating P53. Importantly, the P53-mediated stress response is elicited only if the nucleolar function is affected [97] (Figure 2b).

Nucleolar and ribosomal proteins play a central role in the regulation of the P53-mediated stress response pathway. Nucleophosmin (NPM1 or B23) is a nucleolar protein with diverse functions, including ribosome biogenesis, histone chaperoning and centrosome duplication (reviewed in [103]). NPM1 can regulate the P53-HDM2 axis by its interaction with ARF. ARF (p19^ARF^ for human, p14^ARF^ for mice) is a tumor suppressor transcribed from an alternative reading frame (ARF) of the p16INK4a cyclin-dependent kinase inhibitor; ARF disrupts P53-HDM2 complex by its interaction with HDM2. Under physiological conditions, NPM1 interacts constitutively with ARF, which is then sequestered in the nucleolus. Upon stress, activation of NPM1 releases ARF which in turn translocates to the nucleoplasm, where it interacts with HDM2 to destabilize the P53-HDM2 complex and eventually activate P53 [104]. NPM1 itself has been reported to directly bind either HDM2 or p53 to regulate the HDM2-p53 pathway [105].

Nucleolin (NCL) also modulates the HDM2-P53 axis. NCL is a multifunctional nucleolar protein which participates in rDNA chromatin remodeling through its activity as histone chaperone of H2A-H2B, is essential for basal transcription factors loading and RNA Pol I progression and contributes to pre-rRNA processing (reviewed in [103]). The multifunctionality of NCL is due to its tripartite structure (contains three distinct functional domains) and its ability to shuttle between the nucleus and the cytoplasm. NCL interacts with both HDM2 and P53 [106,107] to regulate P53 stability. Upon stress, NCL translocates to the nucleoplasm where it interacts with HDM2, thus promoting HDM2 ubiquitination and degradation and disrupting its interaction with P53. Moreover, upon DNA damage, NCL forms a ternary complex with P53 and HDM2, which recruits the ubiquitin protease HAUSP. HAUSP regulates NCL stability by deubiquination [108], but how these interactions contribute to P53 regulation remain unclear. NCL has also been reported to regulate P53 on the translational level. NCL binds both 5’ and 3’UTR of *p53* mRNA to repress its translation. DNA damage releases ribosomal proteins from the ribosomes, resulting in increased levels of ribosomal protein RPL26 which disrupts the NCL interaction with *p53* mRNA, allowing for its translation [109].

### Nucleolar Proteins in DNA Repair

Besides their role in the stress response, NCL and NPM1 emerge also as players in repair of damaged DNA outside the nucleolus. NCL interacts with Rad51, the key recombinase of HR-mediated DSB repair; NCL inhibition sensitizes cells to DSBs induced by the topoisomerase II inhibitor amsacrine [110]. Moreover, both NCL and NPM1 are part of the switch recombinase complex SWAP of B-lymphocytes, suggesting a contribution of these nucleolar proteins in recombination repair [111]. DNA DSBs generated by exposure of cells to IR or to genotoxins, e.g., camptothecin, induce NCL translocation to the nucleoplasm [106,112], where it colocalizes with DSB repair proteins [113]. When NCL is depleted from cells treated with genotoxic agents, DSB signaling and repair factors, such as the MRN complex, H2Ax, ATM and MDC1, are recruited at sites of damage but no DSB resolution occurs, indicating a defect at later stages of repair [113,114]. During DSB repair, the nucleosome at the damaged site is disrupted by eviction of histones H2A-H2B and H3-H4, to enable access of the repair proteins to DNA. In NCL depleted cells, H2A-H2B remains in place, indicating that NCL mediates H2A-H2B mobilization from chromatin to the nucleoplasm [113,114]. In support, NCL facilitates H2A-H2B removal from transcriptional sites by the chromatin remodeling complexes SWI/SNF and ACF, both of which are also involved in DSB repair [115,116]. Moreover, NCL interacts with WRN inside the nucleolus, whereas upon IR- or camptothecin-induced DSBs, both translocate at damaged sites in the nucleoplasm where they re-associate. Their interaction inhibits the helicase but not exonuclease activity of WRN in vitro, but how this may contribute to repair remains elusive [112].

Besides NCL, several lines of evidence support an emerging role for NPM1 in DSB repair. Unlike NCL, NPM1 does not massively translocate to the nucleoplasm upon induction of DNA DSBs. A minor population though is phosphorylated at residue T199 and subsequently recruited to ubiquitin conjugates present at DSBs [117]. In vivo expression of a T199A non-phosphorylatable mutant of NPM1 did not compromise DSB foci formation or the recruitment of DNA repair factors, but similar to NCL, resulted in failure of clearance of DSBs at 6 h post IR [117]. NPM1 has been reported to hold chaperone activity with histones H3–H4 as well as H1 and H2 [118,119,120], suggesting that, together with NCL, it could contribute to nucleosome eviction at DSB sites.

By participating in DNA repair outside the nucleolus, nucleolar proteins might as well serve as signals for switching on (nucleolar disruption and protein translocation) and off (sequestration back to the nucleolus, which is then reformed) the stress response.

## 6. rDNA Damage and Disease Onset

The emerging role of rDNA damage in disease onset supports the notion that rDNA damage accumulation may be causal to accelerated aging (progeria) and/or age-related diseases. At present, the impact of rDNA damage replicative senescence and aging has been extensively studied in yeast. For instance, the ribosomal gene copy number is critical for yeast lifespan and is tightly maintained by a mechanism that involves Fob1p, Sir2p and the replication fork barriers (RFBs). RFBs are found downstream of the pre-rRNA coding sequences [121] and are considered rDNA recombination hot spots [122]. Fob1 binding to an RFB [123] mediates a DSB at the stalled fork [124,125] which is repaired by HR [126] and is regulated by the histone deacetylase Sir2p and its target, non-coding promoter E-pro at the intergenic spacer [127]. When the ribosomal gene copy number is below the wild type dosage, Sir2p is inactivated allowing E-pro to be transcribed. E-pro transcripts remove cohesins that hold sister chromatids together, thus promoting unequal pairing and resulting in copy number increase. In case the rDNA copy number is close to wild type levels, Sir2p represses E-pro transcription, cohesins stay in place and equal sister chromatid recombination takes place without altering the copy number. Aberrant intra-chromosomal recombination results in extra-chromosomal rDNA circles (ERCs). Fob1p, Sir2p and ERCs have been shown to determine yeast lifespan: deletion of *FOB1* increases rDNA stability and lifespan [45,128], *SIR2* mutants accumulate ERCs and show reduced life span [45] and ERCs accumulate preferentially in the aging mother cell [129,130]. Thus, it has been suggested that ERCs are toxic byproducts that act as pro-senescence effectors. Recently, Kobayashi has proposed that inactive rDNA copies are essential for suppressing DNA damage response [131] and that rDNA loss itself activates DDR which in turn causes senescence in a ribosomal biogenesis-independent manner [132,133]. Interestingly, yeast strains with low copy rDNA, where most of the repeats are transcriptionally active, show increased sensitivity to DNA damage, e.g., UV irradiation [132].

It is widely accepted that DNA damage accumulation is associated with aging, evident by the accelerated aging syndromes, many of which are caused by inborn DNA repair defects. Thus, the notion that rDNA instability may causally trigger progeria gains scientific interest. Recently, RFBs have been identified in mouse and human rRNA genes [134]. Mammalian SIR2 homologue is not known to be involved in nucleolar DNA integrity, though Sirtuin 7 resides in the nucleolus and activates RNA polymerase I [135,136]. Almost 30% of rDNA repeats in normal individuals exist as palindromic rather than tandem repeats and are probably non-functional. Their frequency goes up to 50% in individuals with Werner syndrome (WRN) progeria; WRN patients carry inborn defects in the WRN RecQ helicase; the latter implies that altering of the fine balance between canonical and palindromic repeats may lead to premature aging phenotypes [67]. It remains, however, to be found whether such palindromic repeats are equivalent to the yeast ERCs. BLM and ATM are also candidates for linking aging to rDNA maintenance. Cells derived from Bloom syndrome (mutated BLM) or Ataxia Telangiectasia (mutated ATM) patients show high variability of the rDNA copy number due to mitotic recombination in relation to their wild type counterparts [137,138]. Moreover, recent evidence suggests reduction of ribosomal DNA repeats as a trait of human aging [139,140]. Age-related neurological disorders, such as Hodgkin’s disease [141] and Parkinson’s disease [142], show deregulated rDNA transcription and nucleolar dysfunction. rDNA copy number variability has been associated with neurodegeneration: increased number was detected in patients with dementia with Lewy bodies [138] and elevated repeats of the 18S rDNA locus combined with increased silent chromatin marks in Alzheimer’s disease patients [143].

The association of nucleolar function with cancer was established over a hundred years ago, when it was observed that cancer cells have large and abnormal nucleoli [144]. Abnormal morphology is in large attributed to hyperactivation of rDNA transcription required to sustain the high metabolic and proliferation rate of cancer cells [145]. Cancer cells are also characterized by rDNA instability, as manifested by increased mitotic instability observed in more than half of lung and colorectal adult cancer samples relative to surrounding normal tissue or peripheral blood of each patient [66]. Bloom syndrome and Ataxia telangiectasia patients also present with high risk of cancer incidence, though this could be acquired to generalized genomic instability.

### DNA Repair Factors Regulating rDNA Transcription and Disease

Deregulation of rDNA transcription is associated with disease: hyperactivation is manifested in cardiovascular disease [146] and cancer, while downregulation is a trait of premature aging syndromes and age-related neurological diseases, e.g., Hodgkin’s disease and Parkinson’s disease. Besides their role in genome maintenance, BLM, WRN and NER factors all interact with RNA Pol I and are all causal to progeria and growth retardation. BLM and WRN both belong to the RecQ subfamily of ATP dependent 3’–5’ DNA helicases, participate in replication and repair of DSBs and positively regulate rDNA transcription. WRN localization in the nucleolus is tightly connected to active rDNA transcription: in quiescent cells, or cells treated with transcription blocking agents, WRN localizes to the nucleoplasm, while upon RNA Pol I reinitiation WRN shuttles back to the nucleolus [147,148]. WRN deficient fibroblasts show decreased levels of rRNA transcription. Exogenously expressed wild type WRN recovers rRNA transcription through interaction of WRN with RNA Pol I [147]. Moreover, WRN mediates promoter clearance and vascular endothelial growth factor (VEGF), fibroblast GF-β (FGF-β) and epidermal GF (EGF) stimulation of RNA Pol I [149]. BLM localization to the nucleolus is also positively associated with ongoing rDNA transcription. BLM deficient cells also show reduced rRNA transcription. BLM interacts with the RNA Pol I subunit RPA194 and possibly participates together with DNA topoisomerase I in unwinding the RNA:DNA hybrids that form in the rDNA and inhibit ongoing transcription [150,151].

Patients with defects in the GG-NER or TC-NER sub-pathway, manifest with strikingly heterogeneous clinical outcomes, ranging from cancer to progeria and developmental or metabolic abnormalities. The latter argues for NER factors having additional roles beyond DNA repair. Indeed, NER factors are now known to function in the regulation of gene expression in human cells [152], the transcriptional reprogramming of pluripotent stem cells [153] and the fine regulation of growth genes during murine development [154,155]. Patients with Cockayne syndrome (defective in *Csb* or *Csa*) and Trichothiodystrophy (defective in Xpb or Xpd) manifest with progeria and mental retardation. Cells isolated from patients baring mutations in *Csb*, *Xpb* or *Xpd* or cells transiently knocked down for CSB show reduced rRNA synthesis [156,157]. Inside the nucleolus, CSB interacts with RNA Pol I, TFIIH subunits XPB and XPD, XPG endonuclease and basal RNA Pol I-associated transcription factors to activate rRNA transcription [156]. Moreover CSB, XPB and XPD are implicated in positive regulation of RNA Pol I elongation [158,159]. A recent study suggests a possible role for CSB in rDNA transcription. rDNA tends to form G quadruplexes (G4) that block ongoing transcription. In CSB deficient cells, nucleolar transcription is hampered, leading to mitochondrial dysfunction. CSB can resolve unimolecular G4 structures in vitro and recombinant CSB rescues the mitochondrial dysfunction of CSB deficient cells [160].

## 7. Conclusions

**Figure 3:**
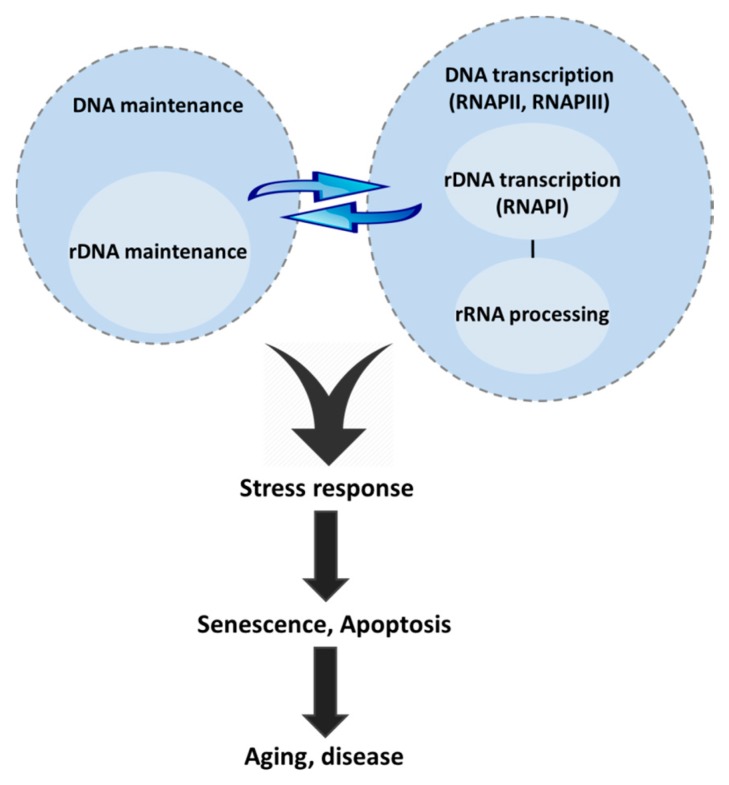
The crosstalk between the DNA and rDNA maintenance pathways and rRNA biogenesis regulates the stress response. DNA damage dramatically affects the nucleolar architecture and function through a network of interactions and translocations where rRNA biogenesis factors participate in DNA repair. Vice versa, DNA maintenance factors participate in and regulate rDNA transcription. In addition, nucleolar factors directly regulate the stress response. Genome maintenance pathways, nucleolar transcription and rRNA processing form a complex network that converges to the stress response, activation of which ultimately leads to aging and/or age-related disease or progeria, whereas evasion leads to cancer.

The nucleolus is well recognized as a central cellular hub for sensing stress stimuli and coordinating stress response. The functional interplay of genome maintenance and ribosomal biogenesis pathways is just emerging as a new player in age-related diseases and premature aging syndromes (Figure 3). DNA damage causes dramatic changes in nucleolar architecture, which is accompanied by protein translocation from nucleoli to the cytoplasm and *vice versa*. Shuttling of nucleolar proteins between the nucleolus and the cytoplasm might also serve as another way of nucleolar sensing of DNA damage and, subsequently orchestrating a response. Moreover, DNA repair proteins that reside in nucleoli to maintain rDNA stability and regulate rRNA transcription offer another level of ribosomal control and disease onset. Premature aging syndromes present with segmental progeria instead of the global functional decline observed in aging. Progeria and a number of age-related diseases show tissue specific aberrant nucleolar function, implying cell type dependent requirements for proper nucleolar function. We do not know though whether nucleolar dysfunction is the direct cause or an effect of disease. The use of in vivo tagging technologies and tissue specific knock out animal models as well as targeting or excluding proteins from the nucleolus, if possible, may contribute to understanding this intertwined network and suggest new therapeutic strategies targeting the nucleolus.
